# Scraps

**Published:** 1895-03

**Authors:** 


### SCRAPS

PICKED UP BY THE ASSISTANT-EDITOR.

**The Means to the End.**—Husband: "I feel completely prostrated. I wish I was dead!" Wife: "Well, why don't you let me send for a doctor?"

**Clinical Records** (10).—Fond mother: "Oh, doctor, we've had such a fearful fright! We thought Johnnie had swallowed a sovereign." Doctor: "And are you sure he hasn't?" Fond mother: "Quite sure, it was only a shilling."

**General Education and Medical Training.**—Editors are always lamenting that before their contributors' communications can be given to the printer, a considerable amount of time is required for the correction of errors of grammar and spelling. It ought not to be necessary to say that those who write for the press should take as much care as possible that no revision of style or composition should be required after their papers leave their hands. Some authorities, however, tell us that in the case of doctors there has often not been the preliminary training which can ensure so desirable a result.

Dr. George M. Gould, the editor of the *Medical News*, is qualified to speak on this subject. In a paper on "The Duty of the Community to Medical Science," he says: To take a boy so untrained that he can't spell correctly any five Anglo-Saxon words, and after a few months' lecture-taking and mnemonic cramming to place in his hands the awful responsibility of the life and health of hundreds or thousands—surely this is a farce worthy only of our civilized barbarism. To illustrate, let me show you a printed sheet containing a student's notes on the differential diagnosis of four varieties of tumors. It needs to be remembered that the student making the notes was about to graduate in 1893, and that the school in which he studied makes much of its preliminary entrance examinations. The young man was an amateur printer. He set up the type himself, corrected the proof, and himself printed off a number of copies for the use of his classmates.

ENCEPHALOID.

1 Soft elastic not uniformly so

2 Groth Rapid Large

3 Adhesions, Earley and Slight

4 Pane, Wandering until ulceration then fixed and servere

5 Veins Large

6 Foul ulcer fungating edges excurvaled undermined much bleding

7 Glandular envolvement early

7½ Comes at any age

8 Seat, Breast, Testicles, Uteris, Overies, Prostate and Salivery glands.

9 Duration 9–12 m fatal.

10 Nipple not retracted.

11 Histary Bad.

SCHIRRUS.

1 Hard, inelastic.
2 Groth, Moderate and Small.
3 Adhesions, Late.
4 Pane, Earley and sharp fixed.
5 Veins, Modatly large
6 Edges, Hard thickened abrupt little bleeding.
7 Glandular envolvement, Late.
7½ after 45X
8 Seat, breast uterus stomach rare in overies and testies and Prostat
9 Fatal 18–36 mts.
10 Retracted nipple.
11 History good, excema of nipple may precid it Pagits diseas.

SARCOMA.

1 Firm, generally irregular soft and flucuating apperently.
2 Rapid and Slow, Groth large size.
3 Adhesions early.
4 Pane slight until, ulceration then more
5 Veins moderate.
6 Foul ulcer, great bleeding,
7 Glandular envolvement late.
7½ Any age.
8 Seat connective tisue extremities of bones periosteum, brest.
9 Long duration before fatal
10 Nipple not retracted.
11 History good.

ADENOMA.

1 Hard elastic.
2 Groth slow.
3 Adhesions rare.
4 Pane neuralgic and menstral.
5 Veins not enlarge
6 No Bleading
7 No glandular envolvement.
7½ Under, 30
8 Seat, brest.
9 Not fatal.
10 Nipple not retracted.
11 History good

**Medical Philology** (XIII.).—The *Catholicon* gives "**Brostyn̄**; *herniosus*. **A Brostynes**; hernia." *Brostynes* was a variant of Burstness, which for centuries was used as a term for hernia. Mr. Herrtage's notes are:

"*Herniosus*. He that is burste or hath his bowells fallen to his coddes. *Hernia*. The disease called bursting." Cooper. Lyte, in his edition of Dodoens, 1578, tells us, p. 87, that "the decoction of the leaues and roote [of the Common Mouse eare] dronken, doth cure and heale all woundes, both inward and outward, and also *Hernies*, *Ruptures*, or *burstings;*" and again, p. 707, that "the barke [of Pomegranate] is good to be put into the playsters that are made against *burstinges*, that come by the falling downe of the guttes." "*Hernia*. Bolnyng of the bowaylles. *Herniosus*. Brostyn." Medulla. Cotgrave mentions a plant, "*Boutouner*. Rupture-wort, Burst-wort." "*Hernia*, broke-ballockyd." Wright's Vol. of Vocab., p. 177.
"*Hernia*, burstnesse." Stanbridge, *Vocabula*.

The *Promptorium* contains "**Brostyn** man, yn the cod. *Herniosus*."

Florio, in his Italian Dictionary (*A Worlde of Wordes*, 1598), gives "**Hernie**, a kind of swelling or inflammatition in a mans cods or stones, but properlie the bursting or rupture of a man when the bowels fall into the cods." Minsheu's *Guide into the Tongues*, 1625, has "**Búrstnesse**, the bowels being fallen into the Cods, making them as the cod of a Bull." In the English and French addition to Cotgrave (1632) is "**Burstnesse**, Rompure, rupture; hérgne, greveure, hernie, hydrocele." "Hergne" and "hernie" are given by Cotgrave as "Bursting; or a rupture within the cods." "Greveure" is defined as "An inward rupture or bursting (of the lower part of the bellie) or an extraordinarie swelling of the cods, by the bowels falling into them, a bursting, or being burst," and "hernė" as "Burst; hauing a rupture about the Priuities." In Beaumont and Fletcher's *Scornful Lady* (v. 3), Savil, speaking to Young Loveless, says of his eldest boy:

"He was born bursten; and, your worship knows,
That is a pretty step to men's compassions."

"Bursten" appears in the additon to Cotgrave as "grevé, hergneux, hernė."

The inclusion of hydrocele in the definition in Cotgrave shows that there was confusion of terms as well as of diagnosis. The term rupture was employed in reference to hydrocele, in language which was not merely popular. The well-known surgeon, Percival Pott, in 1762 published *Practical Remarks on the Hydrocele, or Watry Rupture*. A copy of this is in the Bristol Medical Library.

It is strange that Hernia (the etymology of which is uncertain) and Bursting or Rupture should, unless qualified by some further description, have become almost exclusively used for one surgical affection. It is much the same with Fistula, which has no special significance. This has led more than one person into error. For instance, the king in *All's Well that Ends Well* is represented as suffering from a fistula (I. i. 39). Many people have too hastily concluded that the fistula was an anal one. Here was something which Mr. Bowdler did not understand, and of course he was offended by it. He made short work of it, and cut out the passage. Dr. William Bodenhamer (*New York Medical Journal*, Feb. 10th, 1894, p. 177), in an article on "Incidents in the History of Anal Fistula," says: "Shakespeare has rendered anal fistula quite notorious by making it a very important feature in his play, *All's Well that Ends Well*." And even so well informed a person as G. B. S., writing (*Saturday Review*, Feb. 2nd, 1895, p. 151) upon a performance of *All's Well that Ends Well*, evidently considers that a fistula-in-ano was the complaint which Helena succeeded in curing by means of a prescription which her father had left her. There can be little doubt that Shakspere took the story of the play from Painter's *Palace of Pleasure*, in which the passage from Boccaccio's *Decameron*, containing the mention of the king's malady is thus translated from the Ninth Novel of the Third Day: "The Frenche Kyng had a swellyng upon his breast, whiche by reason of ill cure, was growen to a Fistula, and did putte him to marveilous paine and grief." The labour of expurgation and the squeamishness that have been exercised on this passage have therefore been quite wasted.

The plant which Cotgrave mentions is "Boutonnet," not "Boutouner" as quoted by Mr. Herrtage. Burst-wort is given in the *New English Dictionary* as "an old name for *Herniana glabra*, a herb formerly thought helpful for curing ruptures." In the addition to Cotgrave its equivalents are "Herbe au Turc, hermole, herniaire."

